# Potential pitfalls in diagnosis of immunotherapy-induced hypothalamic–pituitary–adrenal axis suppression

**DOI:** 10.1530/EO-22-0069

**Published:** 2022-08-09

**Authors:** Darran Mc Donald, Rachel Katherine Crowley

**Affiliations:** 1 Department of Endocrinology, St Vincent’s University Hospital, Dublin, Ireland; 2 Department of Medicine, University College Dublin, Dublin, Ireland

We thank Bi and colleagues for their recent case report ‘Potential pitfalls in diagnosis of immunotherapy-induced hypothalamic–pituitary–adrenal axis abnormalities: a clinical case’ (Bi *et al.* 2022). It provides important lessons on the investigation and management of immunotherapy-related hypophysitis, which is an evolving area within endocrinology and oncology. As part of our work, in a hospital whose oncology service is the Irish national referral centre for several malignancies, we manage ever-increasing numbers of immunotherapy-related hypophysitis and would like to share some further observations.

The authors state that the Society for Endocrinology and French Endocrine Society guideline recommend performing a short synacthen test (SST) “if an early morning cortisol is between 138 and 500 nmol/L, to diagnose ‘latent’ adrenal insufficiency” (Bi *et al.* 2022). It is important to clarify that the guideline’s recommendation refers solely to cases of adrenalitis and not hypophysitis (Castinetti *et al.* 2019). We do agree with the author’s opinion that the guidance on the investigation of adrenocorticotrophic hormone (ACTH) deficiency in suspected cases of immunotherapy-related hypophysitis is ambiguous and therefore wish to outline our experience with reference to the relevant literature. Firstly, detailed knowledge of the natural history of immunotherapy-related hypophysitis and a low threshold of suspicion are required to diagnose acute ACTH deficiency, given the non-specific presenting symptoms. We believe that endocrinologists with a particular interest in either pituitary or adrenal endocrinology are best placed to do this. It is important to be aware of specific risk factors for immunotherapy-related hypophysitis and ACTH deficiency, which have largely been identified from retrospective reviews. For instance, combination therapy with a CTLA-4 (ipilimumab) and PD-1 inhibitor (nivolumab) is associated with a higher risk of immunotherapy-related hypophysitis at 10.5% and earlier onset compared with monotherapy (Castillero *et al.* 2019). ACTH deficiency is the most commonly affected anterior pituitary hormone in those receiving anti-CTLA-4 treatment, followed by thyroid-stimulating hormone (TSH) and gonadotrophins with deficiency rates of 91, 84 and 83%, respectively (Caturegli *et al.* 2016). Preserved ACTH secretion in the setting of TSH and gonadotropin deficiency is rare in immunotherapy-related hypophysitis. It has also been noted that ACTH deficiency is commonly preceded by a progressive reduction in TSH (Faje 2016). The accumulation of these findings in the presented case makes the presence of ACTH deficiency exceedingly likely. Given this high degree of suspicion, in the event of an indeterminate morning cortisol level (138–500 nmol/L), we would recommend empirical hydrocortisone with reassessment of adrenal reserve by either measuring a morning cortisol (while holding hydrocortisone) or, very rarely, undertaking an SST after 6 weeks. It is particularly important to empirically prescribe hydrocortisone for those with suspected ACTH deficiency, who are acutely unwell, to prevent an adrenal crisis. Where a low suspicion for ACTH deficiency exists, frequent monitoring of morning cortisol levels may be undertaken in selected patients. In such cases, detailed advice should be given to patients and their families about the symptoms of ACTH deficiency and when to seek medical assistance.

In our clinical practice, we never undertake SSTs to assess ACTH deficiency in the acute setting. It takes approximately 6 weeks from the onset of ACTH deficiency for the zona fasciculata of the adrenal cortex to atrophy. Synacthen can elicit a cortisol response from the adrenal cortex in this period, producing a false negative result. Although the quoted guideline (Castinetti *et al*. 2019) does not recommend performing SSTs to assess ACTH deficiency in the acute setting, we have encountered several review articles that do. This recommendation is in contrast to established endocrine practice in neurosurgical patients. Following pituitary surgery, those with an indeterminate morning cortisol level are empirically prescribed hydrocortisone followed by a reassessment of their adrenal axis at least 6 weeks later (Pofi *et al.* 2019). As there are no studies examining the role of SSTs in the assessment of ACTH deficiency in immunotherapy-related hypophysitis, we must rely on data from neurosurgical patients. In a retrospective review of 62 patients who had a normal SST 1 week post transsphenoidal resection, 23 patients subsequently went on to develop adrenal insufficiency over 1–3 months (Klose *et al.* 2005). Furthermore, three patients presented with symptomatic hypoadrenalism prior to reassessment of their adrenal axis. Therefore, undertaking an SST in the acute setting of ACTH deficiency gives an unacceptably high risk of a false negative result and exposes the patient to the risk of symptomatic hypoadrenalism and adrenal crisis. Insulin tolerance tests (ITTs) are the gold standard in diagnosing ACTH deficiency; they assess the entire hypothalamic–pituitary–adrenal (HPA) axis and therefore are accurate in the acute phase of ACTH deficiency. However, ITTs induce symptomatic hypoglycaemia and require intensive monitoring and repeated blood sampling. For these reasons, we believe it is an unnecessary test to undertake in individuals with advanced malignancies, which is typically the indication for immunotherapy.

Another important consideration in the investigation of immunotherapy-related hypophysitis is the concomitant use of steroids, either as part of a chemotherapy regimen or for palliative symptom control. Exogenous steroids may mask hypophysitis initially while supressing the HPA axis. Withdrawal of steroids after prolonged use can then lead to adrenal insufficiency. It also must be emphasised if there is any suspicion of ACTH deficiency, thyroxine should never be commenced or the dose increased due to the risk of precipitating an adrenal crises.

Magnetic resonance imaging (MRI) of the pituitary can aid the diagnosis of immunotherapy-related hypophysitis. Radiological abnormalities, although not specific, have been reported in 80% of cases and can precede the onset of hormone deficiencies (Albarel *et al.* 2019). It is also used to exclude other causes of pituitary dysfunction such as metastasis, apoplexy, and abscesses. The timing of an MRI is crucial and should be performed at the earliest opportunity as abnormalities can rapidly resolve, giving a falsely normal result. For example, in a small review by Faje *et al.*, radiological abnormalities were resolved in all 7 patients within 40 days (Faje *et al*. 2014). Additional tests such as DHEAS can indicate the adequacy of the HPA axis. A low age- and sex-adjusted DHEAS is a sensitive but not specific marker for ACTH deficiency, meaning a normal DHEAS level makes the diagnosis unlikely but a low value will require further evaluation.

It would be interesting to know whether the patient was hyponatraemic during their period of illness. It has been reported that hyponatraemia occurs in 56% of patients with immunotherapy-related hypophysitis (Faje 2016); however, the proportion of hyponatraemia caused by ACTH deficiency is unknown. Hyponatraemia in this setting is caused by increased levels of corticotropin-releasing hormone which stimulates vasopressin, leading to water retention (Raff *et al.* 1987). Cortisol deficiency also increases the expression of aquaporin-2 channels in the collecting duct of the kidneys which increases free water reabsorption. If hyponatraemia is present, it is mandatory to perform a morning cortisol as ACTH deficiency shares an identical biochemical signature to syndrome of inappropriate diuresis (SIAD), both characterised by raised urinary sodium and osmolality (Mc Donald *et al.* 2022). Differentiating between these two causes of hyponatraemia is important as ACTH deficiency is readily treatable with hydrocortisone and negates the need for arduous fluid restriction (Garrahy & Thompson 2019).

There is growing evidence on the prognostic significance of developing immunotherapy-related hypophysitis; it confers both improved tumour response to therapy and overall survival. In a retrospective review of 228 malignant melanoma patients treated with ipilimumab, median overall survival was 21.4 months in those who developed hypophysitis compared with 9.7 months in those who did not (Faje *et al.* 2016). Although these studies are typically limited by their retrospective nature and sample studies, the increasing number of studies from a variety of malignancies and immunotherapy regimens are encouraging. The potential mechanisms underlying this association are still unknown. Case reports, such as the one outlined, help to inform clinicians and stimulate discussion about challenging cases (Mc Donald *et al.* 2021) in an evolving area of endocrinology. Future guidelines need to address the diagnostic difficulties encountered in immunotherapy-related hypophysitis more comprehensively.

## Declaration of interest

The authors declare that there is no conflict of interest that could be perceived as prejudicing the impartiality of this letter.

## Funding

This work did not receive any specific grant from any funding agency in the public, commercial, or not-for-profit sector.
